# Electrocardiographic QRS Findings Upon Admission Can Predict Prognosis of Acute Myocardial Infarction Caused by Occlusion of Left Main Coronary Artery

**DOI:** 10.7759/cureus.36435

**Published:** 2023-03-20

**Authors:** Osamu Sasaki, Hideki Sasaki

**Affiliations:** 1 Internal Medicine, Kouiki Mombetsu Hospital, Mombetsu, JPN; 2 Cardiology, Saitama Medical Center, Saitama Medical University, Kawagoe, JPN; 3 Cardiovascular Surgery, Nagoya City University East Medical Center, Nagoya, JPN

**Keywords:** bradycardia, qrs duration, electrocardiography (ecg), left main trunk, acute myocardial infarction

## Abstract

Background: Acute myocardial infarction (AMI) caused by left main coronary artery (LMCA) occlusion is associated with a severe clinical course and catastrophic consequences.

Hypothesis: We sought to clarify ECG predictors of prognosis in AMI caused by LMCA occlusion.

Methods: We examined 20 consecutive patients with AMI caused by LMCA occlusion that was treated by primary stenting. The patients were assigned to either a group that survived (S) and was discharged from hospital, or a group that did not survive (NS) and died in hospital. We compared ECG findings upon admission, angiographic findings, laboratory data and clinical outcomes.

Results: The rate of having Thrombolysis In Myocardial Infarction (TIMI) grade > 2 coronary flow before PCI and of achieving TIMI grade 3 after PCI was significantly lower in the NS than the S group (14.3% vs. 83.3%, p = 0.003 and 35.7% vs. 100%, p = 0.008). The ECG findings showed longer QRS interval in the NS than in the S group (150.5 ± 37.9 vs. 105.2 ± 15.4, p = 0.022). A QRS interval ≥ 120 msec predicted in-hospital mortality with sensitivity, specificity and positive and negative predictive values of 78.5%, 100%, 100% and 66.7%, respectively, in this population.

Conclusions: The QRS duration upon admission was a good predictor of in-hospital mortality among patients with AMI caused by LMCA occlusion. This ECG sign could be useful in the emergency clinical setting.

## Introduction

Acute myocardial infarction (AMI) caused by occlusion of the left main coronary artery (LMCA-AMI) usually presents as extensive ischemia of the left ventricle and malignant ventricular arrhythmias that result in severe circulatory failure and collapse. Despite recent developments in percutaneous coronary intervention (PCI) and emergency coronary artery bypass grafting (CABG), treating LMCA-AMI remains challenging and the prognosis is unsatisfactory.

Previous studies have found that ST elevation in lead aVR is a useful predictor of LMCA obstruction and the important determinants of the prognosis were hemodynamic instability, dominant right coronary artery (RCA), intercoronary collateral blood flow and a partially occluded LMCA [1-6]. However, electrocardiographic signs responsible for the prognosis of LMCA-AMI have not been defined. The present study derives useful prognostic predictors from 12-lead electrocardiograms (ECG) obtained from patients with LMCA-AMI upon admission.

## Materials and methods

Study population

We enrolled 20 (1.7%) patients with partially or totally occluded LMCAs among 1180 with AMI treated with primary PCI at our institution between December 2004 and October 2012. None of the patients had received previous CABG. The patients were assigned to either a survivor (S) group that was discharged from hospital, or a non-survivor (NS) group who died in hospital. This retrospective observational study was approved by the institutional review board at Saitama Medical Center, Saitama Medical University (approval 882).

Definitions

The definition of AMI followed the ESC/ACCF/AHA/WHF Task Force for the Universal Definition of Myocardial Infarction [7]. Cardiogenic shock was diagnosed based on systolic blood pressure <80 mmHg without vasopressor or intraaortic balloon pumping (IABP) support, end-organ hypoperfusion with pulmonary congestion (Killip class >2), persistent oliguria (<30 mL/h), clouded consciousness and cold extremities.

Dyslipidemia was defined as low-density lipoprotein cholesterol (LDL-C) level >140 mg/dL, high-density lipoprotein cholesterol (HDL-C) level <40 mg/dL, triglycerides >150 mg/dL, or under cholesterol-lowering medication. Smoking was defined as current smoking or smoking cessation less than one month before enrolment. Hypertension was defined as systolic blood pressure >140 mmHg and/or diastolic blood pressure >90 mmHg or medication with antihypertensive drugs. Patients with diabetes mellitus had a confirmed diagnosis or were medicated with insulin or oral hypoglycemic agents at the time of enrollment.

ECG evaluation

Upon admission to Emergency, 12-lead ECG data were obtained as soon as possible and the following parameters were compared between the groups: heart rate, heart rhythm, QRS width, QRS axis, QTc (corrected QT) interval (QTc interval was measured according to Bazzet`s formula: QTc = QT divided by RR root), ST-segment elevation/depression, abnormal Q waves, inverted T waves and conduction disturbances such as atrioventricular block and accelerated idioventricular rhythm (AIVR). The QRS duration was measured with a caliper in the lead with the longest QRS duration. Deviation of the ST segment could not be evaluated in the presence of a bundle-branch block (BBB). Therefore, ST-segment elevation was assessed in leads aVR and aVL as described [6,8,9]. ST-segment deviation was measured at 60 ms after the J point and significant ST-segment change was defined as >0.05 mV of deviation from baseline.

Angiographic evaluation and PCI procedure

All patients underwent emergency coronary angiography and angiographic coronary blood flow was evaluated using Thrombolysis in Myocardial Infarction (TIMI) flow grades at the beginning and end of each procedure [10]. The presence of angiographic collaterals and predominance of the right coronary artery (RCA) were also assessed. Collateral filling was classified on a scale from 0 - 3 as no visible filling of any collateral channel, filling of side branches via collateral channels without visualization of the epicardial segment, partial filling of the epicardial major coronary artery via collateral channels and complete filling of the epicardial major coronary artery via collateral channels, respectively. After thrombectomy for the LMCA culprit lesion, catheter interventions such as standard balloon angioplasty and primary stenting proceeded. Angiographically successful PCI was defined as residual stenosis of <30% with TIMI grade 3 flow. An initial bolus of unfractionated heparin (3,000 units) was administered followed by 5,000 units immediately before PCI, and continuous infusion for three days. Dual antiplatelet therapy using aspirin and ticlopidine or clopidogrel were continued in all patients. All angiograms were evaluated by the consensus of two independent interventional cardiologists who were blinded to the study protocol and the backgrounds of the patients.

Hospital course and follow-up

We assessed in-hospital clinical events including death, repeat infarction, repeat PCI and emergency CABG. We compared the numbers of days that IABP and/or percutaneous cardiopulmonary support (PCPS) were required between the groups. All patients who were discharged from the hospital were regularly followed up as outpatients.

Statistical analysis

All data were analyzed using SPSS software version 22.0 (IBM Corp., Armonk, NY, USA). Continuous variables are expressed as means ± SD and categorical variables as numbers and ratios (%). Continuous variables were compared using an unpaired t-test or the Mann-Whitney test and categorical variables were assessed using the chi-square test. Multivariate analysis was not considered because of the small sample size. Kaplan-Meier survival curves were prepared and receiver operating characteristic curves were generated to determine the predictive values of the variables. P < 0.05 was considered statistically significant.

## Results

Baseline characteristics of study patients

Table 1 shows the baseline characteristics of the patients. The mean age was 64.5 ± 11.5 (range, 41 - 83) years and 16 (80%) patients were male. Seventeen (85%) of them were in cardiogenic shock upon arrival. Only one of the survivors had a history of myocardial infarction.

**Table 1:** Patients’ characteristics. HDL-C, high density lipoprotein; LDL-C, low density lipoprotein; MI, myocardial infarction.

	All patients (n = 20)	Non-survivors (n = 14)	Survivors (n = 6)	p
Age (y)	64.5±11.5	62.8±12.7	67.1±7.6	0.453
Male	16(80%)	12(85.7%)	4(66.7%)	0.329
Hypertension	15(75%)	9(64.3%)	6(100%)	0.091
Dyslipidemia	15(75%)	9(64.3%)	6(100%)	0.091
Diabetes Mellitus	5(25%)	3(21.4%)	2(33.3%)	0.573
Smoking	8(40%)	6(42.9%)	2(33.3%)	0.690
LDL-C (mg/dL)	94.5±36.6	93.0±37.8	97.4±37.7	0.822
HDL-C (mg/dL)	37±12.9	35.8±12.5	39.1±14.4	0.624
HbA1c (%)	5.9±1.1	5.7±0.9	6.3±1.4	0.292
Prior MI	1(5%)	0(0%)	1(16.7%)	0.117
Prehospital cardioversion	3(15%)	2(14.3%)	1(16.7%)	0.891
Shock upon arrival	17(85%)	13(92.9%)	4(66.7%)	0.132
Killip classification 3/4	16(80%)	11(78.6%)	5(83.3%)	0.807

Electrocardiographic findings

**Figure 1:**
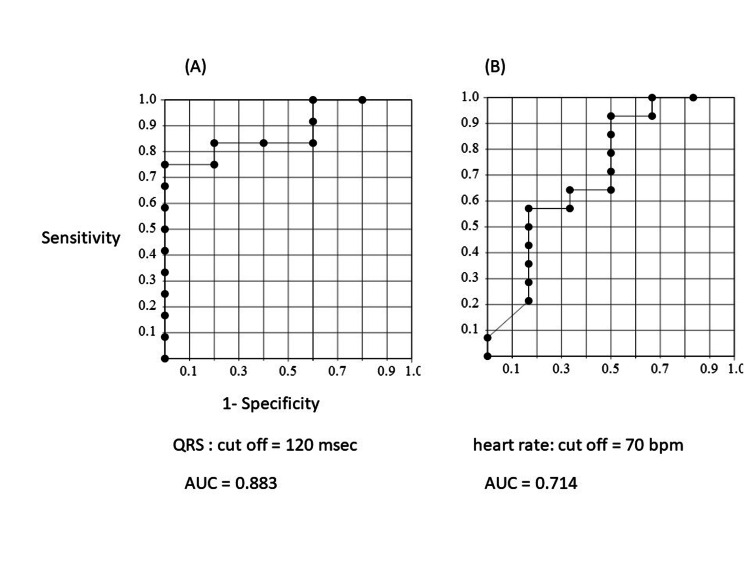
Receiver-operating characteristics curves for predicting in-hospital death according to QRS interval (A) and heart rate (B). Receiver-operating characteristics curves show sensitivity and specificity for predicting in-hospital death according to QRS duration and heart rate. AUC, area under the curve.

**Figure 2:**
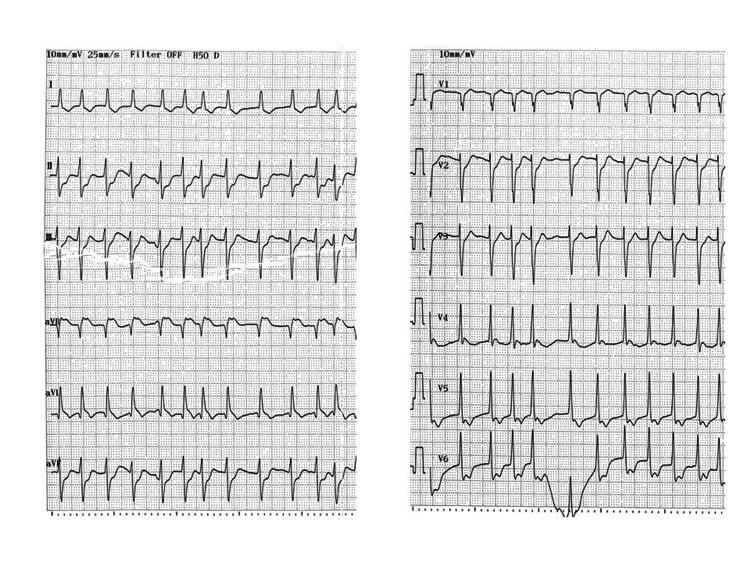
Representative 12-lead electrocardiographic findings of survivors upon admission. Survivor: QRS interval, 90 msec; HR, 137 bpm.

**Figure 3:**
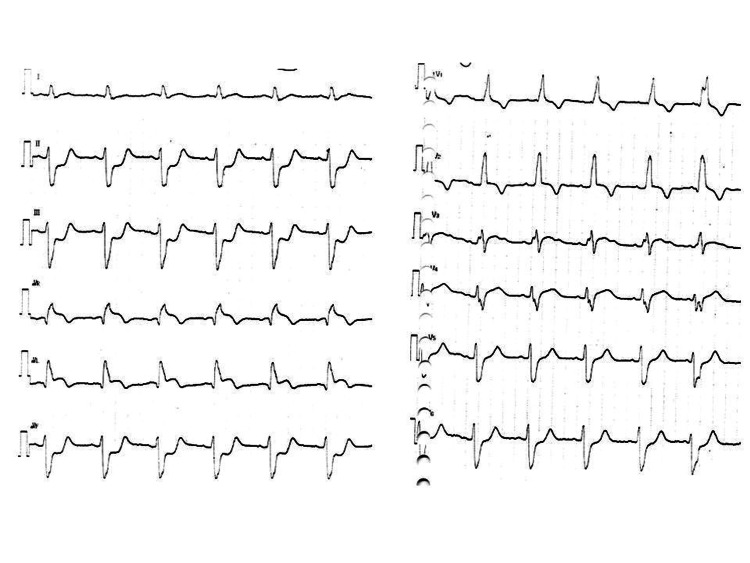
Representative 12-lead electrocardiographic findings of non-survivors upon admission. Non-survivor: QRS interval, 140 msec; HR, 66 bpm. HR, heart rate.

Table 2 shows electrocardiographic findings on admission. Heart rate was relatively slower in the NS than in the S group (78.1 ± 28.7 vs. 103.8 ± 38.7, p = 0.115) and complete right bundle branch block (CRBBB) was more frequent in the NS than in the S group (71.4% vs 16.7%, p = 0.024). Figure 1 shows the sensitivity and specificity for predicting in-hospital mortality according to QRS interval. The cut-off value for the QRS interval with the optimal sensitivity and specificity was 120 msec by the closest-to-(0, 1) criterion [11]. A QRS interval ≥120 msec predicted in-hospital mortality with a sensitivity, specificity and positive and negative predictive values of 78.5%, 100%, 100% and 66.7%, respectively, in this population. The cut-off heart rate required for optimal sensitivity and specificity was 70 beats/min. Figures 2, 3 show representative 12-lead electrocardiograms from the S and NS groups.

**Table 2:** ECG findings upon admission. CLBBB, complete left bundle branch block; CRBBB, complete right branch block; dep, depression; HR, heart rate; inf, inferior leads; QTc, corrected QT (Bazett's formula).

	All patients (n = 20)	Non-survivors (n = 14)	Survivors (n = 6)	p
HR (beats/min)	85.8±33.2	78.1±28.7	103.8±38.7	0.115
HR<70 bpm	9(45%)	8(57.1%)	1(16.7%)	0.095
CRBBB	11(55%)	10(71.4%)	1(16.7%)	0.024
CLBBB	2(10%)	1(7.1%)	1(16.7%)	0.515
QRS width (ms)	137.1±38.7	150.5±37.9	105.2±15.4	0.022
QRS axis (°)	-27±55.9	-30.3±65.4	-19.0±25.5	0.716
QTc interval (ms)	474.2±73.1	468.2±70.2	488.6±86.4	0.617
QRS ≥120 (ms)	11(55%)	11(78.6%)	0(0%)	0.001
ST elevation in aV_R_	12(60%)	7(50%)	5(83.3%)	0.163
ST elevation in aV_L_	14(70%)	11(78.6%)	3(50%)	0.201
ST dep in inf leads	13(65%)	7(100%)	6(100%)	n/a
Abnormal Q wave	17(85%)	12(85.7%)	5(83.3%)	0.891
Inverted T wave	15(75%)	9(64.3%)	6(100%)	0.091

Angiographic findings

Table 3 shows significantly more patients with pre-PCI TIMI flow grade 0, significantly fewer patients with pre PCI TIMI flow grades ≥2 (64.3% vs. 16.7%, p = 0.051 and 14.3% vs. 83.3%, p = 0.003) and significantly fewer post PCI TIMI grade 3 flow (35.7% vs. 100%, p = 0.008) in the NS, the S group. Intercoronary collateral flow, predominant RCA, and the frequency of PCPS and/or IABP requirements did not significantly differ between the groups. Table 4 shows the coronary artery lesion and ECG findings. There is no significant difference in ECG findings between patients with LMCA lesion alone and those with both LMCA and RCA lesions.

**Table 3:** Angiographic findings and clinical course. CABG, coronary artery bypass grafting; CK, creatine kinase; IABP, intraaortic balloon pumping; LOS, low output syndrome; MOF, multiple organ failure; PCI, percutaneous coronary intervention; PCPS, percutaneous cardiopulmonary support; RCA, right coronary artery; TIMI, thrombolysis in myocardial infarction.

	All patients (n = 20)	Non-survivors (n = 14)	Survivors (n = 6)	p
Lesion Location				
ostial	8(40%)	6(42.9%)	2(33.3%)	0.799
body	9(45%)	7(50%)	2(33.3%)	0.492
distal	3(15%)	1(7.1%)	2(33.3%)	0.132
Left coronary stenosis > 75%				
Left anterior descending	2(10%)	1(7.1%)	1(16.7%)	0.515
Circumflex artery	4(20%)	3(21.4%)	1(16.7%)	0.807
Right coronary artery stenosis >75%	7(35%)	5(35.7%)	2(33.3%)	0.918
Right coronary artery occlusion	0			
Pre PCI TIMI = 0	10(50%)	9(64.3%)	1(16.7%)	0.051
Pre PCI TIMI ≥2	7(35%)	2(14.3%)	5(83.3%)	0.003
Post PCI TIMI3	11(55%)	5(35.7%)	6(100%)	0.008
Collateral grade ≥2	2(10%)	1(7.1%)	1(16.7%)	0.515
Dominant RCA	5(25%)	4(28.6%)	1(16.7%)	0.573
Peak CK (U/L)	12872.7±9395.6	16636.2±8617.4	4091.1±3191.3	0.003
PCPS applied	14(70%)	11(78.6%)	3(50%)	0.201
PCPS weaned	17(85%)	11(78.6%)	6(100%)	0.218
PCPS (days)	3.2±2.6	3.9±2.6	1.6±1.8	0.07
IABP applied	16(80%)	11(78.6%)	5(83.3%)	0.807
IABP weaned	16(805%)	10(71.4%)	6(100%)	0.143
IABP (days)	5.5±4.7	6.5±5.2	3.0±1.7	0.123
Subacute thrombosis	1(5%)	1(7.1%)	0(0%)	0.501
Septicemia	11(55%)	9(64.3%)	1(16.7%)	0.051
MOF with LOS	14(66.7%)	14(100%)	0(0%)	<0.001
CABG at chronic phase	1(5%)		1(16.7%)	n/a

**Table 4:** Coronary artery lesion and ECG findings. AIVR, accelerated idioventricular rhythm; AV, atrioventricular; CLBBB, complete left bundle branch block; CRBBB, complete right branch block; HR, heart rate; LMCA, left main coronary artery; QTc, corrected QT (Bazett's formula); RCA, right coronary artery.

	LMCA alone (n=13)	LMCA+RCA (n=7)	p
Sinus rhythm	6(46.2%)	5(71.4%)	0.278
Complete AV block	1(7.7%)	0	0.452
AIVR	4(30.8%)	1(14.3%)	0.416
HR (beats/min)	76.7±35.1	97.2±28.2	0.270
HR<70 bpm	7(53.8%)	2(28.6%)	0.278
CRBBB	8(61.5%)	3(42.9%)	0.423
CLBBB	1(7.7%)	1(14.3%)	0.639
QRS width (ms)	135.3±30.3	141.2±58.7	0.792
QRS axis (°)	-10.3±52.7	-67.0±45.3	0.054
QTc interval (ms)	467.1±82.8	491.4±44.8	0.549
QRS ≥120 (ms)	8(61.5%)	3(42.9%)	0.423

In-hospital outcomes

**Figure 4:**
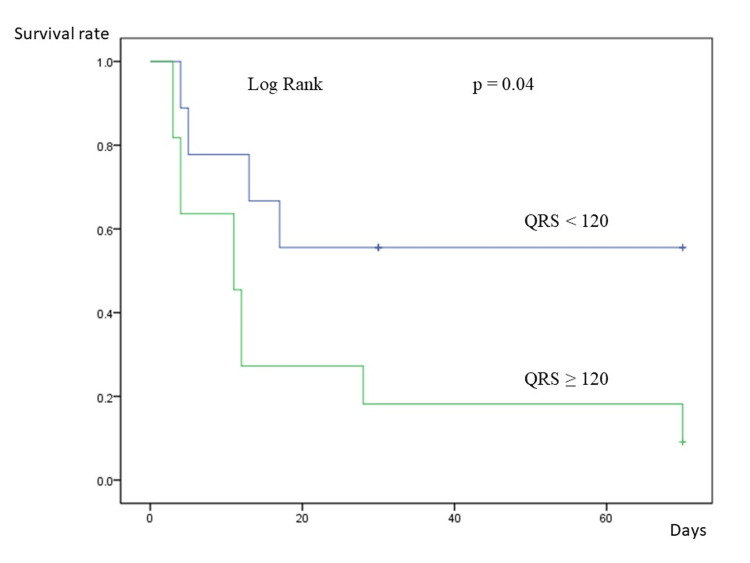
In-hospital survival of patients stratified by QRS interval. QRS interval ≥120 msec predicts in-hospital survival.

The maximum creatine kinase (CK) value was significantly higher in the NS than in the S group (16636.2 ± 8617.4 U/L vs. 4091.1 ± 3191.3 U/L, p = 0.003). The duration of PCPS was longer for the NS than the S group (3.9 ± 2.6 days vs. 1.6 ± 1.8 days, p = 0.07). Fourteen (67.7%) patients died in hospital, nine (64.3%) non-survivors presented with septic shock with low output syndrome, one patient developed subacute stent thrombosis on day seven after admission, and all patients in the NS group died of multiple organ failure with low output syndrome. Figure 4 shows Kaplan-Meier survival curves during hospitalization. Having QRS ≥120 msec predicted in-hospital mortality.

Long-term outcomes

One patient in the S group underwent elective CABG for unstable angina with angiographic restenosis six months after discharge. The overall survival rate was 30.0% and no late deaths occurred in the S group during a mean follow-up of 4.5 ± 3.3 years.

## Discussion

Clinical presentations of AMI caused by LMCA lesions are usually catastrophic and most patients with LMCA-AMI die due to out-of-hospital cardiac arrest or sudden cardiac death before reaching a hospital. Studies have shown that some factors such as hemodynamic instability, pre-interventional TIMI flow grade, intercoronary collaterals and incomplete occlusion of the LMCA are related to prognosis. However, ECG findings related to prognosis remain relatively unknown. We showed that ECG findings can help to simply discriminate between survival and in-hospital death.

Electrocardiographic findings

The ECG findings of the LMCA-AMI might depend on the degree and extent of myocardial ischemia and be influenced by residual blood flow in the LMCA, intercoronary collateral flow, right or left dominance of the coronary artery and other anatomical features.

Previous studies have found variable and dissimilar ECG patterns in LMCA-AMI including ST elevations in aVR and other leads, and widespread ST depression with inverted T waves in limb and chest leads [5-6]. In ST elevation AMI, ST elevation in the aVR is considered an effect of transmural ischemia of the basal part of the septum. Lead aVR looks into the left ventricular cavity from the right shoulder. In non-ST elevation AMI, ST elevation in aVR reveals global subendocardial ischemia [12]. Most patients in our population presented with ST elevation in aVR and ST depression in inferior leads. These findings are considered to indicate extensive myocardial damage.

One report [13] found a significantly longer QRS interval on ECG from patients with AMI caused by lesions of the LMCA than of the left anterior descending coronary artery, the left circumflex coronary artery and the RCA. A long QRS interval suggests extensive damage of the myocardium, such as an intraventricular conduction delay and interrupted depolarization caused by excessive global ischemia or infarction. However, to the best of our knowledge, only one report has described differences in ECG findings between survivors and non-survivors with LMCA-AMI [6]. That study found more frequent ST elevations in the aVR and aVL leads in non-survivors than in survivors (83% vs. 17%). We found ST elevations in both of these leads in all patients who died and in half of those who survived (100% vs. 50%), and although the NS could be discriminated from the S group with 100% sensitivity, the specificity was only 50%. In contrast, the QRS was significantly broader in the NS than in the S group and a QRS interval ≥120 msec predicted in-hospital mortality with sensitivity and specificity of 78.5% and 100%, respectively. A wider QRS interval indicated massive myocardial damage and more extensive damage to the intraventricular conduction system in the NS than in the S group.

Tachycardia is a normal compensatory response that is rather common in patients with shock. However, the heart rates in most of the non-survivors in this study did not increase regardless of cardiogenic shock, which further worsened hypoperfusion of the tissue and the organs of the whole body. Such paradoxical hemodynamics have been termed, “relative bradycardia” to describe a discrepancy between body temperature and heart rate in patients with infectious diseases and as ‘pulse-temperature differences’ [14-15]. The heart rates of eight (57.1%) patients in the NS group and one (16.7%) in the S group were <70 beats/min despite remarkable hypotension. All patients in the NS group presented with shock upon arrival, and heart rates that were inappropriate responses to circulatory collapse. This situation might also be caused by conduction disturbances due to extensive ischemia of the myocardium including the conduction system.

Clinical outcomes

Septicemia with low output syndrome developed in nine (64.3%) patients in the NS group while in hospital and the NS group needed PCPS and/or IABP longer than the S group. Although mechanical support such as IABP and PCPS were necessary for life support, long-term use of these devices might have caused infection.

According to previous reports, hemodynamic instability, pre-interventional TIMI flow grade, intercoronary collaterals and subtotal occlusion of the LMCA have been considered important factors related to prognosis in patients with LMCA-AMI [1-4]. Pre-PCI TIMI flow grade 0 was significantly more frequent and post-PCI TIMI grade flow 3 was significantly less frequent in the NS than in the S group. These findings were consistent with previous reports. Furthermore, the long-term outcomes of our patients who were discharged from the hospital were excellent as in previous reports [3-4].

Previous studies have found that an increasing QRS duration predicts higher mortality rates for patients with AMI, heart failure and out-of-hospital cardiac arrest and indicate that a wide QRS interval reflects more extensive damage to the conduction system [16-18]. A prolonged QRS is also associated with asynchronous ventricular contraction and prognosis [19-20]. Our results were consistent with these reports and increasing QRS interval predicted in-hospital outcomes. We consider that QRS widening indicating extensive and profound circumferential ischemia of the entire heart, an extreme reduction in wall motion and a conduction disturbance at the time of presentation to hospital subsequently resulted in poor in-hospital outcomes.

Limitations

The retrospective study at a single tertiary care center has several limitations.

A large number of survivors of out-of-hospital cardiac arrest were transferred to this facility. Prior ECG findings were unavailable and thus were not evaluated. Although prehospital factors such as time from onset to hospital arrival and echocardiographic parameters on admission are important predictors of outcomes in the emergency clinical setting, we lack data on both of these parameters. Specifically, we do not have information on the time required for hospital arrival and the echocardiographic parameters were not adequately evaluated and recorded during the acute phase. Our study population did not undergo any surgical procedures during the acute phase. A previous study found that CABG in LMCA-AMI resulted in a poor prognosis with a perioperative mortality of 46% and the authors suggested the importance of intensive myocardial protection and aggressive postoperative circulatory support [21]. Medical therapy and PCI might not be the optimal strategy to achieve desirable outcomes for patients with a wide QRS interval on ECG. Advanced mechanical support including a left ventricular assist device, a total artificial heart or heart transplantation might be required [22], and regenerative therapy might be a future option.

## Conclusions

The simple electrocardiographic finding of QRS duration ≥120 msec upon admission was a powerful predictor of a poor prognosis for patients with LMCA-AMI, who were treated by emergency PCI. Standard PCI seems insufficient to achieve better outcomes and new treatment strategies are needed for this patient population.
